# Modeling count data for health care utilization: an empirical study of outpatient visits among Vietnamese older people

**DOI:** 10.1186/s12911-021-01619-2

**Published:** 2021-09-15

**Authors:** Duc Dung Le, Roberto Leon Gonzalez, Joseph Upile Matola

**Affiliations:** 1 National Graduate Institute for Policy Studies (GRIPS), 7-22-1 Roppongi, Minato-ku, Tokyo 106-8677 Japan; 2 Institute of Social and Medical Studies (ISMS), No. 810 & 804, CT1A DN1 Building, Ham Nghi St., Nam Tu Liem Dist., Hanoi, 10000 Vietnam; 3 Ministry of Economic Planning and Development, Capital Hill, Lilongwe 3, Malawi

**Keywords:** Count data, Vietnam, Modeling healthcare utilization, Older people, Outpatient visits, Hurdle models, Overdispersed data

## Abstract

**Background:**

Vietnam is undergoing a fast-aging process that poses potential critical issues for older people and central among those is demand for healthcare utilization. However, healthcare utilization, here measured as count data, creates challenges for modeling because such data typically has distributions that are skewed with a large mass at zero. This study compares empirical econometric strategies for the modeling of healthcare utilization (measured as the number of outpatient visits in the last 12 months) and identifies the determinants of healthcare utilization among Vietnamese older people based on the best-fitting model identified.

**Methods:**

Using the Vietnam Household Living Standard Survey in 2006 (N = 2426), nine econometric regression models for count data were examined to identify the best-fitting one. We used model selection criteria, statistical tests and goodness-of-fit for in-sample model selection. In addition, we conducted 10-fold cross-validation checks to examine reliability of the in-sample model selection. Finally, we utilized marginal effects to identify the factors associated with the number of outpatient visits among Vietnamese older people based on the best-fitting model identified.

**Results:**

We found strong evidence in favor of hurdle negative binomial model 2 (HNB2) for both in-sample selection and 10-fold cross-validation checks. The marginal effect results of the HNB2 showed that ethnicity, region, household size, health insurance, smoking status, non-communicable diseases, and disability were significantly associated with the number of outpatient visits. The predicted probabilities for each count event revealed the distinct trends of healthcare utilization among specific groups: at low count events, women and people in the younger age group used more healthcare utilization than did men and their counterparts in older age groups, but a reverse trend was found at higher count events.

**Conclusions:**

The high degree of skewness and dispersion that typically characterizes healthcare utilization data affects the appropriateness of the econometric models that should be used in modeling such data. In the case of Vietnamese older people, our study findings suggest that hurdle negative binomial models should be used in the modeling of healthcare utilization given that the data-generating process reflects two different decision-making processes.

## Background

Healthcare utilization data, such as the number of an individual’s outpatient visits to hospitals, typically manifests as count data (observations that have only nonnegative integer values). This data is usually characterized by a substantial point mass at zero, a long right tail of individuals who make more use of healthcare, and a tendency for the variance to increase with the mean. Such datasets pose significant modeling challenges compared to data that is normally distributed, for instance. Consequently, modeling healthcare utilization has received considerable attention in the field of health economics given how important it is to understand the factors that drive healthcare utilization when making policy. Additionally, the choice of econometric models affects modeling outcomes including the predicted probability of use of healthcare services and the likelihood of being extensive users of such services.

In this paper, several models are considered in our analysis, with the Poisson regression model (PRM), a basic model for count data, taken as a starting point. However, the Poisson distribution has one major shortfall which is inherent in its unique property known as “equidispersion”- a property where the conditional mean and the conditional variance are same. Such a property has been shown to be too restrictive for modeling healthcare utilization given that count variables often have a variance greater than the mean, a condition known as “overdispersion”. One model that addresses this issue is the negative binomial regression model (NBRM). The NBRM has a built-in parameter that accounts for the overdispersion problem thereby making its estimates substantially more efficient than those of the PRM.

Two other regression models considered for count data are the hurdle regression model (HRM) and the zero-inflated regression model (ZIM). The former allows for zeros and positive observations generated by two different processes. In particular, the HRM reflects two different decision-making processes: whether to use healthcare or not; and (conditional on the decision of use of healthcare) how much care to consume. The HRM can be viewed as a principal-agent model, where the principal (the patient) initiates the first visit to a hospital and there together with the agent (the physician) decide on the second and subsequent visits [1].

Unlike the HRM which allows for the possibility that zeros are generated by a different process from positive observations, the ZIM, introduced by Mullahy [2] and Lambert [3], considers the zeros as being generated by two distinct processes namely: structural and sampling processes. The strategy behind the ZIM reflects the intuition that there are two latent groups in the population—potential users and nonusers. For example, in the context of outpatient visits to hospitals, it might be reasonable to think that the population comprises two types of groups—individuals who would never seek outpatient services in hospitals and those who would. There are therefore two possibilities for observing zeros. Either the individual having zero outpatient visits just happened not to seek outpatient services during the survey period (sampling zeros) or would never do so (structural zeros).

Although the HRM and ZIM can both be viewed as two-component finite mixture models, such mixture is of a limited form because the zeros are treated in separate processes in those count models. Another model known as the latent class model (LCM) provides a more general finite mixture model which has powerful properties for the modeling of healthcare utilization. Unlike the HRM and ZIM, the LCM makes no distinction between users and non-users of care. Rather, in a case of two latent sub-populations, it distinguishes two groups as “healthy” and “ill” [4]. The LCM allows for heterogeneity along the outcome distribution by means of complex configurations of either observed or unobserved characteristics. The LCM for unobserved heterogeneity rests on the assumption that the unobserved heterogeneity which divides the population into latent classes is based on individuals’ latent long-term health status. Therefore, population heterogeneity may not be well captured by proxy variables such as self-rated health or chronic health conditions [5]. In using these models, researchers are typically interested in the distinction between extensive margins—zero counts versus positive counts (no outpatient visit versus at least one outpatient visit)—and intensive margins—how many positive counts if nonzero counts (how many subsequent outpatient visits after the first visit is made).

To date, most studies on modeling healthcare utilization using count data have been conducted in developed countries and little is known in the context of the developing world. Given the differences in the healthcare systems and healthcare behavior in the two worlds, results of the studies conducted in developed countries may lack relevance or adaptability for developing countries. In developing countries, studies on healthcare expenditure, particularly those looking at catastrophic payments for healthcare or healthcare payments and poverty, have attracted more attention than those of healthcare utilization measured as count events. To the best of our knowledge, there has been no study on modeling healthcare utilization in developing countries, particularly focusing on older people. This study contributes to empirical evidence on the best choice of econometric models for count data in developing countries.

The study aims to: (i) identify the model that best explains variability in the number of outpatient visits by comparing empirical econometric strategies for the modeling of healthcare utilization; and (ii) identify the determinants of healthcare utilization among Vietnamese older people based on the results of the best-fitting model identified. The study examines the effectiveness of the PRM, the NBRM, the HNB model and its extensions, the ZIM and its extensions and the LCM.

## Institutional background

In line with a rapid demographic transition towards an aging society in the world, Vietnam is undergoing a fast aging process and is expected to experience the fastest aging process in Southeast Asia region [6]. Sturdy economic growth since *Doi Moi* (economic renovation) in the late 1980s has resulted in considerable improvement in socio-economic status and the healthcare system in Vietnam. In Vietnam, life expectancy at age 60 is relatively high, with an expected 25 and 19 more years for women and men, respectively [7]. However, these extra years consist of an average of seven years living with illness/disability for women and five years for men [7], leading to a rise in demand for healthcare utilization among older people. Particularly, healthcare utilization among Vietnamese older people is mostly outpatient visits (91.0% for governmental, private and other health institutions combined) [7]. Social health insurance (SHI) in Vietnam was introduced in 1992, followed by a series of reforms, to provide individuals with access to healthcare services and to reduce out-of-pocket (OOP) spending in fee-for-service. As a result, SHI coverage among older people was significantly increased from 43.5% in 2006 to 75.0% in 2014 and OOP was reduced over time [7].

## Methods

### Data

The data used in this paper was drawn from the 2006 Vietnam Household Living Standard Survey (VHLSS), conducted by the Vietnam General Statistics Office. VHLSS, similar to the Living Standard Measurement Study, is one of the most commonly used household surveys in developing countries. VHLSS is conducted every two years and the information collected is used to assess living standards of populations in all regions and localities across the country. The survey gathers data on a variety of topics such as demographic characteristics of household members, household income, household expenditure, education, health, employment, assets, housing facilities, and participation in hunger elimination and poverty reduction. The VHLSS sampling framework developed in 2006 is based on that of the 1999 Viet Nam Housing Population Census. A three-stage stratified design method was adopted for the survey sampling.

To date, among waves of the VHLSS conducted, the 2006 VHLSS contained the richest information on the health conditions—disability and non-communicable diseases (NCDs)—and lifestyle—smoking of household members. The final sample size of the 2006 survey was 45,945 households including an income survey of 36,756 households and expenditure survey of 9189 households. At household level, the survey collected information on household income, household expenditure and household size. At the individual level, various information on individual characteristics was collected including age, gender, ethnicity, education, marital status, working status and health conditions. In this study, older people (defined as those aged 60 and older) were of interest, so we restricted our analysis to a sample of 2624 people without missing values for our variables of interest.

### Measurement of variables

#### Count outcome variable

**Fig. 1:**
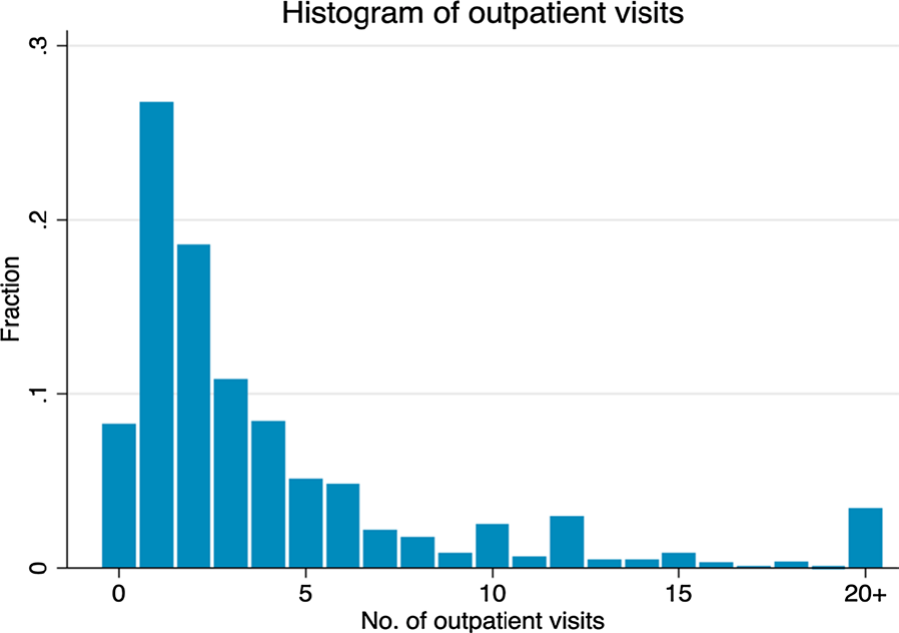
The frequency distribution of outpatient visits

The count outcome variable was the number of outpatient visits in the last 12 months. Summary statistics and frequency distributions are reported in Table 1 and Fig. 1, respectively.

**Table 1:** Definition of the selected variables

Variable	Definition	Mean	S.D
Outpatient visits	Number of outpatient visits in the last 12 months	4.336	6.437
Age	Age in years	71.465	7.977
Sex	Male = 0; female = 1	0.599	0.490
Ethnicity	Non-Kinh people = 0; Kinh people = 1	0.894	0.307
Household size	Log of household size	1.251	0.567
Place of residence	Rural = 0; urban = 1	0.262	0.439
Red River Delta	= 1, otherwise = 0	0.233	0.423
East Northern Mountainous areas	= 1, otherwise = 0	0.098	0.298
West Northern Mountainous areas	= 1, otherwise = 0	0.024	0.153
North Central Coast	= 1, otherwise = 0	0.098	0.298
South Central Coast	= 1, otherwise = 0	0.122	0.328
Central Highlands	= 1, otherwise = 0	0.044	0.024
Southeast	= 1, otherwise = 0	0.135	0.342
Mekong Delta	= 1, otherwise = 0	0.245	0.430
Marital status	Married = 0; single = 1	0.405	0.491
Social health insurance	No health insurance = 0; has health insurance = 1	0.549	0.497
Health subsidy	No health subsidy = 0; received health subsidy = 1	0.587	0.493
Employment status	Not working = 0; working = 1	0.424	0.494
Education	Education of respondents in years	4.075	3.642
Household income	Log of household income	10.017	0.878
Smoking	Not smoking = 0; smoking = 1	0.323	0.468
Non-communicable diseases (NCDs)	No NCD = 0; having at least one NCD = 1	0.356	0.479
Disability	No disability = 0; having at least one disability = 1	0.285	0.452

S.D. denotes standard deviation

#### Explanatory variables

In selecting explanatory variables, we relied on the conceptual framework of the behavioral model of health services utilization developed by Andersen [8, 9]. Developed to understand individuals’ access to healthcare services and utilization, the model has been widely used in empirical studies to examine determinants of healthcare utilization [10, 11]. The model has shown that individuals’ healthcare utilization could be influenced by three broad groups of factors including predisposing, enabling and need factors [8]. A comprehensive discussion on the behavioral model and how the three groups of factors are classified is presented elsewhere [10]. In addition to these three groups of factors, we also controlled for lifestyle factors since it has been well documented that unhealthy behaviors such as smoking have a negative effect on individuals’ health which in turn might influence healthcare seeking behavior. Detailed information of the four groups of explanatory variables is described below.

Predisposing variables reflect demographic characteristics of respondents and in this study, these included age, age squared, sex, marital status and ethnicity. Enabling variables refer to differences in access to healthcare and in this study, they comprised log of household size, place of residence, region of residence, education, employment status, log of household income, SHI, and health subsidy. Need variables capture the need for healthcare and these consisted of disability and NCDs. Finally, smoking was included as the lifestyle variable. Definitions of the explanatory variables are presented in Table 1.

### Regression models for count data

#### Poisson regression model (PRM)

The PRM assumes that a discrete random dependent variable $y_{i}$, representing the number of times that an event occurs, follows a Poisson distribution with mean $\mu_{i}$ (the expected number of times that the event will occur during a given period of time). The PRM is defined by the density:

1 $$f\left( {y_{i} {|}{\varvec{x}}_{i} } \right) = \frac{{e^{{ - \mu_{i} }} \mu_{i}^{{y_{i} }} }}{{y_{i} !}},\quad for\,\, y_{i} = 0, 1, 2, \ldots ,$$

where the conditional mean is defined as:

$$\mu_{i} = E\left[ {y_{i} {|}{\varvec{x}}_{i} } \right] = \exp \left( {{\varvec{x}}_{i}^{^{\prime}} {\varvec{\beta}}} \right),$$

where ${\varvec{x}}_{i}$ is the vector of covariates, ${\varvec{\beta}}$ is a (*k* × 1) parameter vector of unknown coefficients, and $!$ is the factorial operator [5].

#### Negative binomial regression model (NBRM)

Following Cameron and Trivedi [5], the NB density for a random discrete count outcome *y* = 0, 1, 2, … can be written as:

2 $$f\left( {y_{i} | {\varvec{x}}_{i} } \right) = \frac{{{\Gamma }\left( {\alpha^{ - 1} + y_{i} } \right)}}{{{\Gamma }\left( {\alpha^{ - 1} } \right){\Gamma }(y_{i} + 1)}}\left( {\frac{{\alpha^{ - 1} }}{{\alpha^{ - 1} + u_{i} }}} \right)^{{\alpha^{ - 1} }} \left( {\frac{{u_{i} }}{{\alpha^{ - 1} + u_{i} }}} \right)^{{y_{i} }} \quad for\,\,\alpha > 0,$$

where ${\Gamma }\left( . \right)$ denotes the gamma function and $\alpha > 0$ is a constant dispersion parameter to be estimated. The first two conditional moments of the NBRM are

$$\begin{aligned} & E[y_{i} |{\varvec{x}}_{i} ] = u_{i} = {\text{exp}}\left( {{\varvec{x}}_{i}^{^{\prime}} {\varvec{\beta}}} \right) \\ & V\left[ {y_{i} {|}{\varvec{x}}_{i} } \right] = u_{i} + \alpha u_{i}^{2} . \\ \end{aligned}$$

The above specification corresponds to the most used version of the NBRM known as the negative binomial 2 (NB2). A less used version of the NBRM, termed as the negative binomial 1 (NB1), maintains the same specification of the conditional mean as in NB2 but specifies the conditional variance as linear in the mean.

$$V\left[ {y_{i} {|}{\varvec{x}}_{i} } \right] = u_{i} + \alpha u_{i} .$$

#### Hurdle regression model (HRM)

In the HRM for count data, proposed by Mullahy [12], zero and positive parts can be estimated separately using two different densities: $f_{1} \left( . \right)$ and $f_{2} \left( . \right)$. Specifically, the zero part is determined by $f_{1} \left( . \right)$, such that $\Pr \left( {y_{i} = 0} \right) = f_{1} \left( 0 \right)$, while the positive part determining the amount of healthcare usage is specified by $f_{2} \left( . \right)$, such that the probability of observing *y*, for *y* > 0, is $f_{2} \left( {y_{i} {|}y_{i} > 0} \right) = f_{2} \left( {y_{i} } \right)/\left\{ {1 - f_{2} \left( 0 \right)} \right\}$. In practice, the most common choice for $f_{1} \left( . \right)$ is logit model, which is used here. The typical choice for $f_{2} \left( . \right)$ is usually either a truncated-at-zero Poisson or negative binomial (NB). The probability function of the HRM can be written as:

3 $$\Pr \left( {y_{i} = j{|}{\varvec{x}}_{i} } \right) = \left\{ {\begin{array}{*{20}l} {f_{1} (0{|}{\varvec{x}}_{i} {)}} \hfill & {if\,\,j = 0 } \hfill \\ {\frac{{1 - f_{1} \left( {0{|}{\varvec{x}}_{i} } \right)}}{{1 - f_{2} \left( {0{|}{\varvec{x}}_{i} } \right)}}f_{2} \left( {j{|}{\varvec{x}}_{i} } \right)} \hfill & { if\,\, j > 0,} \hfill \\ \end{array} } \right.$$

where $$f_{1} \left( {0{|}{\varvec{x}}_{i} } \right) = \frac{{{\text{exp}}\left( {{\varvec{x}}_{i} {\varvec{\beta}}} \right)}}{{1 + {\text{exp}}\left( {{\varvec{x}}_{i} {\varvec{\beta}}} \right)}}$$.

For the hurdle Poisson model (HPM) specification, $f_{2} \left( {0{|}{\varvec{x}}_{i} } \right) = \exp \left( { - \mu_{i} } \right) = {\text{exp}}\left( { - {\varvec{x}}_{i}^{^{\prime}} {\varvec{\gamma}}{ }} \right)$ and $f_{2} \left( {j_{i} {|}{\varvec{x}}_{i} } \right)$ is specified as the standard PRM described in Eq. (1). As for the hurdle negative binomial model (HNB), $f_{2} \left( {0{|}{\varvec{x}}_{i} } \right) = \left( {1 + \alpha \mu_{i} } \right)^{ - 1/\alpha }$ and $f_{2} \left( {j{|}{\varvec{x}}_{{\varvec{i}}} } \right)$ corresponds to the NBRM described in Eq. (2). The conditional mean of Eq. (3) is given by:

$$E\left[ {y_{i} {|}{\varvec{x}}_{i} } \right] = \frac{{1 - f_{1} \left( {0{|}{\varvec{x}}_{i} } \right)}}{{1 - f_{2} \left( {0{|}{\varvec{x}}_{i} } \right)}}\mu_{2} \left( {{\varvec{x}}_{i} } \right),$$

where $\mu_{2} \left( {{\varvec{x}}_{i} } \right)$ is the conditional mean of the second part.

#### Zero-inflated regression models (ZIM)

If the probability of being potential non-users is *q*, then (1-*q*) is the probability of being potential users. The probability function of the ZIM can be defined as [13]:

4 $${\text{Pr}}[y_{i} = j|{\varvec{x}}_{i} ] = \left\{ {\begin{array}{*{20}c} {q + \left( {1 - q} \right)f_{2} \left( {0|{\varvec{x}}_{i} } \right) if j = 0 } \\ {\left( {1 - q} \right)f_{2} \left( {j|{\varvec{x}}_{i} } \right) if j > 0, } \\ \end{array} } \right.$$

where $f_{2} \left( . \right)$ is the density of either the PRM or NBRM. In Eq. (4), positive counts arise only from the process that generates users, while zeros arise from both processes. For the zero-inflated Poisson (ZIP) specification, $f_{2} \left( 0 \right)$ = ${\text{exp}}\left( { - \mu_{i} } \right)$ = ${\text{exp}}\left( { - {\varvec{x}}_{i}^{^{\prime}} {\varvec{\gamma}}} \right)$ and $f_{2} \left( {j{|}{\varvec{x}}_{{\varvec{i}}} } \right)$ corresponds to the standard PRM described in Eq. (1). In the case of zero-inflated negative binomial 2 (ZINB2), $f_{2} \left( 0 \right)$ = $\left( {1 + \alpha \mu } \right)^{ - 1/\alpha }$ and $f_{2} \left( {j{|}{\varvec{x}}_{{\varvec{i}}} } \right)$ corresponds to the NBRM described in Eq. (2).

#### Latent class models (LCM)

A random outcome variable, *y,* is drawn from one of *C* distributions, with probability $\pi_{c}$ of being drawn from that distribution, such that 0 $\le$ $\pi_{c}$ $\le$ 1 and $\sum\nolimits_{c = 1}^{C} {\pi_{c} = 1}$. Then, the density function for a *C*-component finite mixture is defined as:

5 $$f\left( {y_{i} {|}{\varvec{x}}_{i} ; \theta_{1} , \theta_{2} , \ldots , \theta_{C} ; \pi_{1} , \pi_{2} , \ldots , \pi_{C} } \right) = \mathop \sum \limits_{c = 1}^{C} \pi_{c} f_{c} (y_{i} |{\varvec{x}}_{i} ; \theta_{c} ),$$

where $f_{c} (y_{i} |{\varvec{x}}_{{\varvec{i}}} ; \theta_{c} )$ are the density for class or component c (c = 1, 2, …, C) and $\theta_{c}$ are the parameters of the distributions $f_{c} \left( . \right)$ [14–16].

Common choices for distributions of count data are the NB, which is used here. The latent class NB2 (LCNB2) model assumes that each of the component distributions follows a NB2 model with mean $\mu_{c,i}$ and overdispersion $\alpha_{c}$. For an individual in class c, the LCNB2 with gamma density for an outcome *y* can be expressed as a density function:

6 $$f_{c} \left( {y_{i} {|}{\varvec{x}}_{i} ; \theta_{c} } \right) = \frac{{{\Gamma }\left( {\alpha_{c}^{ - 1} + y_{i} } \right)}}{{{\Gamma }\left( {\alpha_{c}^{ - 1} } \right){\Gamma }(y_{i} + 1)}}\left( {\frac{{\alpha_{c}^{ - 1} }}{{\alpha_{c}^{ - 1} + u_{c,i} }}} \right)^{{\alpha_{c}^{ - 1} }} \left( {\frac{{u_{c,i} }}{{\alpha_{c}^{ - 1} + u_{c,i} }}} \right)^{{y_{i} }} ,$$

where $\theta_{c} = \left( {\alpha_{c} , \beta_{c} } \right)$ and $\mu_{c,i} = {\text{exp}}\left( {{\varvec{x}}_{i}^{^{\prime}} {\varvec{\beta}}_{{\varvec{c}}} { }} \right)$ [13]. In this model, $\left( {\alpha_{c} , \beta_{c} } \right)$ are unrestricted across latent classes. The expected value of the outcome, $y_{i}$, given covariates ${\varvec{x}}_{i}$, is:

$$E\left( {y_{i} {|}{\varvec{x}}_{i} } \right) = \mathop \sum \limits_{c = 1}^{C} \pi_{c} \mu_{c,i} .$$

### Identification of the best-fitting model

In this section, we describe a three-step procedure used to identify the best-fitting model among the count models considered in the study. First, model specifications were examined using Ramsey’s Regression Specification Error Test (RESET). We then used statistical tests and goodness-of-fit measures for performance evaluation of in-sample selection. Lastly, we conducted 10-fold cross-validation checks to examine reliability of the in-sample model selection. In sum, we defined the best-fitting model as the one whose specification was correctly specified, and performance was the best in terms of model fit for both in- and out-of-sample selections. Details on how each procedure is conducted are provided in the following sections.

### Model specification tests

Deb et al. [14] demonstrated that choosing wrong model specifications might lead to inconsistent estimates of parameters and misleading results. We used Ramsey’s RESET test to examine specification of the explanatory variables in the context of the PRM. In short, the test regresses the dependent variable (here, the number of outpatient visits) on its predicted values and their powers (i.e., the squared, cubed, and fourth-order terms of the predicted values), and test whether the coefficients on the higher-order terms were jointly significantly different from zero. Results of the test provide information on whether important variables correlated with high-order terms were omitted or not [14]. Detailed information on Ramsey’s RESET test can be found in other literature [14, 17]. Results of the test show that our PRM was correctly specified since the test statistic was found to be statistically insignificant at the conventional *p*-value = 0.05 (not shown here).

### In-sample model selection

Two common approaches are used to evaluate performance of count models. The first compares mean predicted probabilities and observed proportions for each count of outpatient visits, while the second uses statistical tests and goodness-of-fit measures for performance evaluation among the count models considered. Using the first approach, we computed average predicted probabilities for counts 0–20, since those count events accommodated most observations of outpatient visits. Then, we compared these estimated probabilities with the corresponding observed proportions of assigned counts in each count model considered. With the second approach, we used likelihood ratio (LR) and Vuong tests for model discrimination among nested and non-nested models, respectively. Basically, the LR test uses − 2 times the difference in the fitted log-likelihoods of the two nested models. Among the selected count models, the NB1 and NB2 were nested within the HNB1 and HNB2, respectively. Similarly, the PRM was nested within either the HPM or the NBRM, and the ZIP was nested within the ZINB2. In addition, the Vuong [18] test was performed to evaluate efficiency among non-nested models. The test is computed as:

7 $$V = \frac{{\overline{{m_{i} }} \sqrt N }}{{s_{{m_{i} }} }},$$

where $m_{i} = \ln \left\{ {\frac{{\widehat{{Pr_{1} }}\left( {y_{i} {|}x_{i} } \right)}}{{\widehat{{Pr_{2} }}(y_{i} |x_{i} }}} \right\}$, $\sqrt N$ is the square root of the sample size; $\overline{{m_{i} }}$ and $s_{{m_{i} }}$ are the mean and standard deviation of $m_{i}$, respectively; $\widehat{{Pr_{1} }}(y_{i} |x_{i} )$ and $\widehat{{Pr_{2} }}(y_{i} |x_{i} )$ are the predicted probability of observing $y_{i}$ in the first and the second models, respectively. The Vuong test asymptotically follows a normal distribution, so the first model is favored if *V* is greater than 1.96 and the second model is favored if *V* is smaller than − 1.96 [18]. Wilson [19] has shown that using the Vuong test to examine performance of the ZIM is invalid, thus we simply used model selection criteria and goodness-of-fit measures for the ZIM.

Regarding model diagnostics, we computed two commonly used model selection criteria: the Akaike information criteria (AIC) [20] and the Bayesian information criteria (BIC) [21], for comparison among the selected count models. The two criteria, found to be robust to model misspecification [22], can be computed as:

8 $$AIC = - 2\ln \left( L \right) + 2k,$$

9 $$BIC = - 2\ln \left( L \right) + \ln \left( N \right)k,$$

where ln(*L*) is the maximized log likelihood and *k* is the number of parameters in the model. Smaller values in both AIC and BIC are preferable.

We also computed measures of goodness-of-fit, measured as root mean square error (RMSE) and mean absolute prediction error (MAPE), to evaluate whether the preferred model provided a good fit for the data. The two measures of goodness-of-fit capture the bias between predicted probabilities and observed proportions for each count of the count models considered. Therefore, the smaller the bias, the better the model. RMSE and MAPE can be computed as:

10 $$RMSE = \sqrt {\frac{{\mathop \sum \nolimits_{i = 1}^{N} \left( {y_{i} - \widehat{{y_{i} }}} \right)^{2} }}{N}} ,$$

11 $$MAPE = \frac{{\mathop \sum \nolimits_{i = 1}^{N} \left| {\left( {y_{i} - \widehat{{y_{i} }}} \right)} \right|}}{N},$$

where $\widehat{{y_{i} }}$ is the predicted probability for each count.

### K-fold cross-validation

A potential drawback of using heavily parameterized models for a given dataset is that such models may be overfitting a particular sample of the data and performing poorly in terms of out-of-sample forecasts. This implies that in-sample model performance may not always be reliable. *K*-fold cross-validation checks provide a useful guide to out-of-sample testing [23, 24]. In *K*-fold cross-validation, the original dataset is randomly divided into *K* sub-datasets of approximately equal sizes. Among the *K* sub-datasets, a single sub-dataset is taken as a validation dataset for model testing, and the remaining *K* − 1 sub-datasets are used as training. It is important to note that each observation in the original dataset is randomly assigned to a single sub-dataset and kept in that sub-dataset during the cross-validation examination. The cross-validation procedure is such that *K*-1 models are first trained using the training datasets, and then the estimates of those models are evaluated on the validation dataset. The cross-validation process is repeated *K* times (the folds), with each of the *K* sub-datasets used exactly once as the validation data. This means that each sub-dataset has a chance to be used one time in the validation part and used to train models *K*-1 times. In practice, there is no formal rule for choosing values of *K,* but the choice of* K* is usually 5 or 10. In this exercise, we used 10-fold cross-validation as is the common practice.

## Results

### Descriptive results

Table 1 presents definitions and summary statistics for variables used in the models. On average, in 2006 a Vietnamese older person used outpatient services approximately four times over that year. The variance of outpatient visits was 6.437^2^ = 41.441, roughly 10 times the mean of 4.336, suggesting that the data was very highly overdispersed relative to the Poisson.

The frequency distribution of outpatient visits is shown in Fig. 1. As the figure shows, the frequency distributions of outpatient visits are truncated at 20 visits, implying that there were some excess zeros. It turns out that the 2006 VHLSS had probability mass concentrated on a few values and was highly skewed to the right tail. In particular, the proportion of 0 to 20 visits accounted for about 97.0% of outpatient visits, most of which were concentrated in the 1 to 6 range (about 83.0%, taken together) while the zero counts accounted for only about 8.0% of the visits. The right tail of distributions of outpatient visit was very long, with a maximum value of 104.

### In-sample model selection

**Fig. 2:**
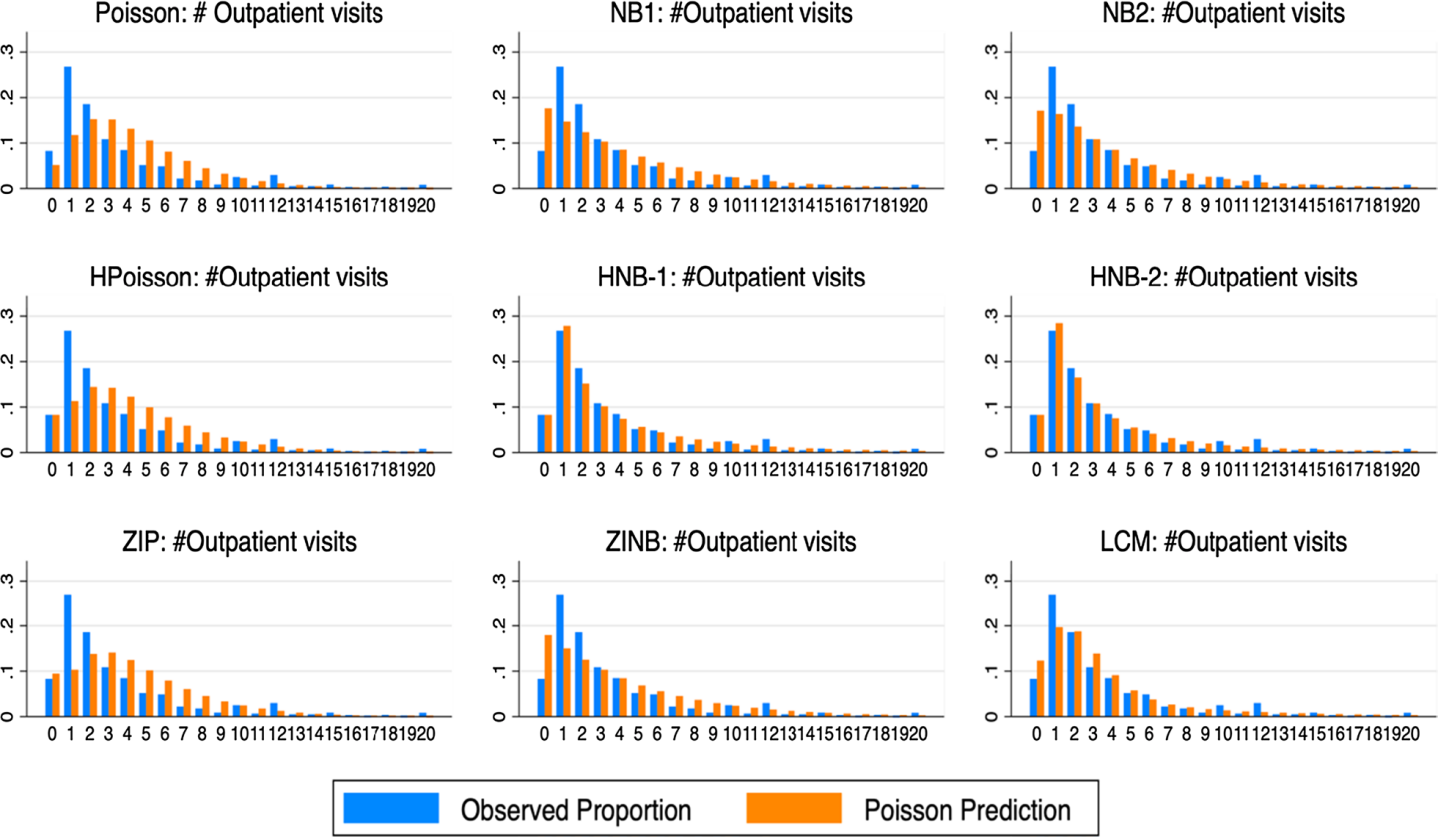
Mean predicted probabilities and observed proportions for each count of outpatient visits among count models. Notes: HNB stands for the hurdle negative binomial, LCM for the latent class model, NB for the negative binomial, ZIP stands for the zero-inflated Poisson, ZINB for the zero-inflated negative binomial, and HPoisson stands for the hurdle Poisson

Figure 2 presents histograms of mean predicted probabilities and observed proportions for 0 to 20 counts of outpatient visits amongst the selected count models. The blue bars depict the actual count frequencies in each count cell, while the orange bars depict the mean predicted probabilities. The figure highlights the extent to which the probability of each count was over- or under-predicted, especially for the zeros. In this study, we used only the NB2 density for the LCM analysis. Also, we did not consider either NB1 density or three-component finite mixture models because they had convergence problems despite our attempts of using built-in options in Stata (such as *difficult* option). In addition, the results of the ZINB2 model should be taken with caution because the number of iterations was limited to 30 due to convergence problems. It can be seen that the HNB1, HNB2, and HPR models produced exactly the same mean predicted probabilities at zero counts as those of the actual frequencies, while other count models over-predicted probabilities of the zero counts, except for the PRM, which was under-predicted. Regarding other count events, the PRM and its hurdle showed a worse fit, while the NBRM and its hurdles showed a better fit relative to the PRM and its hurdle. The LCNB2 and the ZINB2 models also appear to be a better fit than the PRM and its hurdle. Overall, it appears that based on the histograms the HNB1 and HNB2 were the preferred models. However, such visualization simply gives us an overall picture of the selected model performance at each count event. In the following section we further analyze the model selection issues using the information criteria and statistical tests.

In Table 2, results of log likelihood, information criteria and goodness-of-fit measures for each count model considered are summarized. The PRM had the smallest log likelihood values at log(*L*) = 8550, making it the worst model. The HNB2 model had the largest log likelihood values among all the models considered, suggesting that the HNB2 was the preferred model. The results of information criteria also showed strong evidence in favor of the HNB2 model because its values in the AIC and BIC were the smallest among the compared models, followed by those in the HNB1 and LCNB2 models, respectively. The results of goodness-of-fit also favored the HNB2 model, since that model produced the smallest bias between the predicted probabilities and the observed proportions among the count models considered.

**Table 2:** Results of log likelihood, information criteria and goodness-of-fit measures

Model	*K*	Log (*L*)	AIC	BIC	RMSE	MAPE
PRM	34	− 8549.928	17,167.856	17,364.852	1.856	0.531
NB1	35	− 6085.813	12,241.626	12,444.416	1.680	0.433
NB2	35	− 5942.007	11,954.016	12,156.806	1.469	0.372
HNB1	69	− 5751.954	11,641.908	12,041.694	0.491	0.184
HNB2	69	− 5698.735^a^	11,535.471^a^	11,935.257^a^	0.405^a^	0.160^a^
HPM	68	− 8314.001	16,764.001	17,157.993	1.818	0.485
ZIP	68	− 8332.979	16,801.958	17,195.95	1.931	0.518
ZINB2	69	− 6218.109	12,574.219	12,974.005	1.667	0.426
LCNB2	72	− 5769.265	11,682.53	12,099.698	0.905	0.253

PRM stand for the Poisson regression model, NB is the negative binomial, HNB is the hurdle negative binomial, HPM represents the hurdle Poisson model, ZIP the zero-inflated Poisson, ZINB is zero-inflated negative binomial, and LCNB the latent class negative binomial. K is the number of parameters estimated for each model, Log(L) denotes log likelihood, and RMSE and MAPE stand for root mean square error and mean absolute prediction error, respectively

^a^indicates the preferred model

The results of LR tests of the NBRM versus the HNB models, the ZINB2 model versus the ZIP model and the HPR model versus the PRM are presented in Table 3. Here, the NBRM was rejected in favor of the HNB models for both NB1 and NB2 densities. The ZIP model and the PRM were rejected in favor of the ZINB2 and HPR models, respectively.

**Table 3:** Results of the LR tests among nested count models

Pair model	Differences in LR	1% critical value
HNB1^a^ vs. NB1	667.718***	*χ*^2^(34) = 56.1
HNB2^a^ vs. NB2	486.544***	*χ*^2^(34) = 56.1
ZINB2^a^ vs ZIP	4229.739***	*χ*^2^(1) = 6.6
HPM^a^ vs. PRM	471.855***	*χ*^2^nn = 56.1

LR denotes the likelihood ratio, HNB represents the hurdle negative binomial, NB indicates the negative binomial, ZINB means the zero-inflated negative binomial, ZIP denotes the zero-inflated Poisson, HPM represents the hurdle Poisson model, and PRM means the Poisson regression model

^a^indicates the preferred model in pair comparison; and χ^2^(.) means chi-square test and the number in the bracket refers to degree of freedom of each model considered

^*^p < 0.05, **p < 0.01, and ***p < 0.001

Results of the Vuong tests for non-nested models are summarized Table 4. Although we have 10 pairs of non-nested models, however we were particularly interested in comparisons of the HNB2 and other models since because the HNB2 model appears preferable to other count models considered here, based on the results of previous information criteria and LR tests. The Vuong test was also found to favor the HNB2 model with the test statistic for the HNB2 model against the HNB1 model at 3.6, against 12.9 for the HPM, and 4.2 for the LCNB2 model. Furthermore, those test statistics exceeded the critical value of 1.96, suggesting that the HNB2 model was a better fit.

**Table 4:** Results of the Vuong tests among non-nested count models

Pair model	Vuong tests
HNB2^a^ vs. HNB1	3.584
HNB2^a^ vs. HPM	12.883
HNB2^a^ vs. LCNB2	4.233
HNB^a^ vs. HPM	12.848
HNB^b^ vs. LCNB2	0.807
LCNB2^a^ vs. HPM	12.428
LCNB2^a^ vs. NB1	10.043
LCNB2^a^ vs. NB2	9.239
NB^a^ vs. HPM	11.682
NB2^a^ vs. HPM	12.147

HNB denotes the hurdle negative binomial, HPM means the hurdle Poisson model, LCNB2 indicates the laten class negative binomial 2, NB means the negative binomial and vs. denotes versus

^a^indicates the preferred models based on the Vuong test results; and ^b^denotes no evidence of one model is superior to the other

### The 10-fold cross-validation

**Fig. 3:**
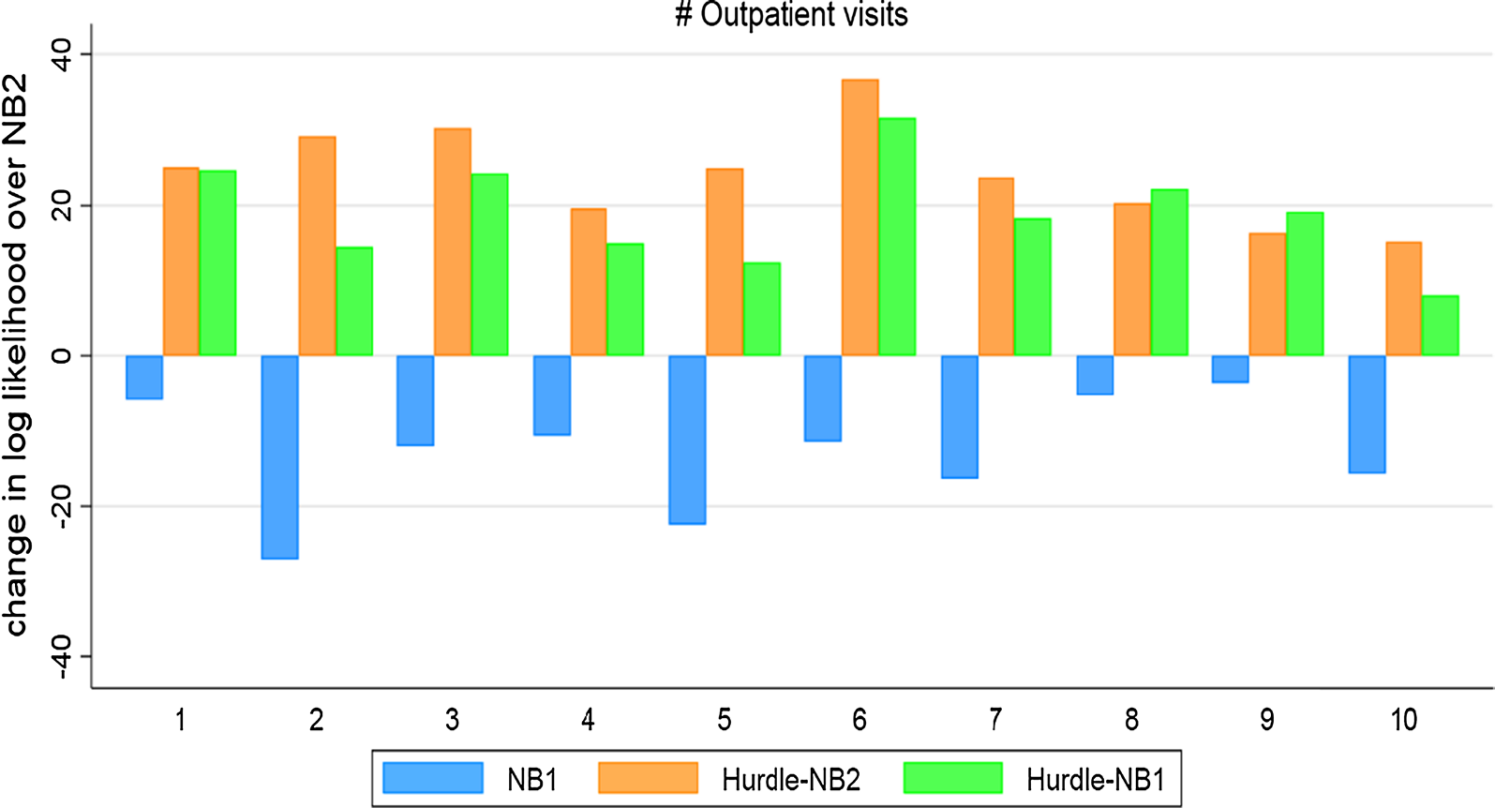
Results of 10-fold cross validation among the NB1, NB2, HNB1, and HNB2. Notes: NB represents the negative binomial and Hurdle-NB denotes the hurdle negative binomial

Figure 3 shows comparison between the NBRM and its hurdles. For ease of interpretation, we do not report the PRM and its hurdle and the ZIM since these models have already been shown to have considerably worse fit than the NBRM and its hurdles. In this exercise, the NB2 was used as the base model. The vertical bars depict the difference in log likelihood of the validation sub-dataset with respect to the NB2 model, while the horizontal bars depict the 10 replications of the selected models. Since the 10-fold cross-validation used log likelihoods to compare models in each replication, a model with the highest log likelihood relative to the NB2 model is preferred. The NB1 model performed worst in each replication, but its hurdle performed better than the NB2 model. Notably, the HNB2 model performed best in 8 out of 10 replications.

### Marginal effects of the best-fitting model

The results of the in-sample model selection and 10-fold cross validation showed that the HNB2 was the best-fitting model. In this section, we computed marginal effects of the HNB2 model to determine the association between the number of outpatient visits and its explanatory variables. Marginal effects from the HRM, as a whole, require putting the part estimating zero counts and the part estimating positive counts together. More specifically, the unconditional rate (meaning both zero and positive counts being estimated, conditional on the explanatory variables) was computed by combining the mean rate for those with zero counts and the mean rate for those with positive counts.

12 $$E\left( {y_{i} {|}{\varvec{x}}_{i} } \right) = \left( {1 - \pi_{i} } \right)*E\left( {y_{i} {|}y_{i} > 0, {\varvec{x}}_{i} } \right),$$

where $E\left( {y_{i} {|}y_{i} > 0, {\varvec{x}}_{i} } \right) = \frac{{\mu_{i} }}{{1 - \left( {1 + \alpha \mu_{i} } \right)^{ - 1/\alpha } }}$ and $\Pr \left( {y_{i} = 0{|}{\varvec{x}}_{i} } \right) = \pi_{i}$. We used the *suest* and the *expression ()* option in *margins* command to obtain overall marginal effects from Eq. (12). The *suest* command provides correct standard errors for the HRM model, since that command takes into account the fact that although the two parts are independently estimated, they are dependent. The results of marginal effects of the HNB2 model are summarized in Table 5.

**Table 5:** The results of marginal effects of the HNB2 as a whole

Variables	Coefficient (S.E.)	*P* value
Age	0.154 (0.20)	0.445
Age square	− 0.001 (0.00)	0.361
Sex (male-reference)		
Female	− 0.685 (0.42)	0.102
Ethnicity (non-Kinh people-reference)		
Kinh people	1.091 (0.385)	0.005
Place of residence (rural areas-reference)		
Urban areas	0.480 (0.381)	0.208
Region of Vietnam (Red River Delta-reference)		
East Northern Mountainous areas	− 0.692 (0.22)	0.002
West Northern Mountainous areas	0.003 (0.68)	0.997
North Central Coast	− 0.226 (0.27)	0.409
South Central Coast	0.322 (0.31)	0.303
Central Highlands	1.218 (0.59)	0.042
Southeast	3.689 (0.64)	0.000
Mekong Delta	4.136 (0.49)	0.000
Marital status (married-reference)		
Single	0.182 (0.24)	0.443
Log household size	− 0.801 (0.35)	0.024
Social health insurance (no-reference)		
Yes	0.581 (0.28)	0.039
Employment status (no-reference)		
Yes	− 0.172 (0.26)	0.517
Education	0.004 (0.05)	0.933
Log household income	− 0.064 (0.30)	0.831
Health subsidy (no-reference)		
Yes	0.591 (0.36)	0.100
Smoking (no-reference)		
Yes	− 0.941 (0.37)	0.012
Non-communicable diseases (no-reference)		
Yes	2.167 (0.24)	0.000
Disability (no-reference)		
Yes	1.158 (0.33)	0.000

HBN2 represents the hurdle negative binomial 2 and S.E. denotes standard errors

The results showed that ethnicity, SHI, NCDs and disability had a significantly positive effect on the probability of visiting hospitals for outpatient services, log household size and smoking had a significantly negative effect, while the region variable produced mixed results. Specifically, the sample average incremental effect of being from the Kinh people was 1.09 meaning that Kinh people averaged 1.09 more outpatient visits than non-Kinh people, with other variables held constant. Similarly, those with SHI had on average 0.58 more outpatient visits than individuals without SHI. Individuals with either NCDs or disability had 2.17 and 1.16 more outpatient visits than those without NCDs and without disability, respectively. As for covariates with negative effects, an additional member of household was estimated to decrease the number of outpatient visits by 0.8, and smokers had 0.94 less outpatient visits than non-smokers.

### Predicted probabilities of using outpatient visits at specific values

Policy-makers and researchers are typically interested in key variables that have strong impact on the health outcomes. Although the findings of this study showed a significant effect of SHI on number of outpatient visits, we are particularly interested in examining the healthcare utilization trend among specific groups. It is possible that healthcare needs could be varied by age and gender. We find predicted probabilities at specific values to be particularly illustrative for interpretation of each count event for specific groups. In this regard, we examined the predicted probabilities of outpatient visits for two groups: those with SHI and those without. In each group, we had six age-gender sub-groups including men aged 60–69, men aged 70–79, men aged 80 +, women aged 60–69, women aged 70–79 and women aged 80 +. After fitting the HNB2, we computed predicted probabilities for each count of each hypothetical group selected. In this exercise, we presented only the predicted probabilities at count 0–10, since estimates of those count events sufficiently showed the healthcare utilization trend among the selected groups. Therefore, the predicted probabilities for those counts may not sum up to one.

**Fig. 4:**
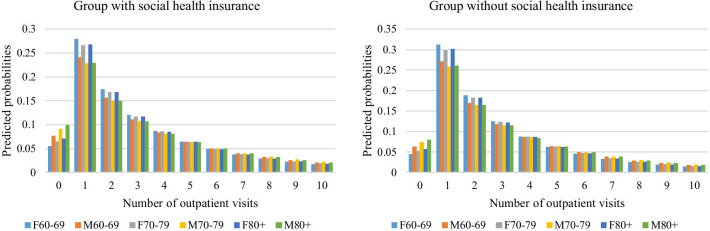
Visualizations of the predicted probabilities at specific count events among groups. Abbreviation: F denotes females and M means males

The predicted probabilities are presented in Table 6 and visualized in Fig. 4. Overall, it can be seen that the probabilities of healthcare utilization decreased when number of outpatient visits increase. This result is reasonable since an individuals’ health could be managed or under controlled after the first or second visit to doctors with probably the exception of severe health conditions. In that case, the probabilities of subsequent visits for them would be diminished. Readers may find that it would be easier to interpret the results for groups with and without SHI if number of outpatient visits were divided into two parts: count 1–4 and count 5–10. The reason is that each part showed the distinct trends of healthcare utilization among the two groups. For the group without SHI at count 1–4, women used more healthcare services than men across age groups, and people in younger age groups had higher predicted probabilities of using healthcare utilization than their counterparts in older age groups, regardless of their gender. By contrast, the results at count 5–10 showed a totally reverse trend as compared to those of count 1–4.

**Table 6:** Results of the predicted probabilities at specific count events among groups

Groups	Number of outpatient visits										
	0	1	2	3	4	5	6	7	8	9	10
Without SHI											
F60–69	0.044	0.312	0.188	0.125	0.087	0.062	0.045	0.033	0.025	0.019	0.014
M60–69	0.063	0.271	0.17	0.118	0.086	0.064	0.049	0.038	0.029	0.023	0.018
F70–79	0.052	0.298	0.182	0.123	0.087	0.063	0.047	0.035	0.026	0.02	0.015
M70–79	0.074	0.258	0.164	0.115	0.085	0.064	0.049	0.038	0.03	0.024	0.019
F80+	0.057	0.301	0.182	0.122	0.086	0.062	0.046	0.034	0.026	0.019	0.015
M80+	0.08	0.26	0.164	0.115	0.084	0.063	0.049	0.038	0.029	0.023	0.018
With SHI											
F60–69	0.055	0.279	0.174	0.12	0.087	0.064	0.049	0.037	0.029	0.022	0.017
M60–69	0.077	0.241	0.156	0.111	0.083	0.064	0.05	0.04	0.032	0.026	0.021
F70–79	0.065	0.266	0.168	0.117	0.086	0.064	0.049	0.038	0.03	0.023	0.019
M70–79	0.091	0.228	0.149	0.108	0.081	0.063	0.05	0.04	0.033	0.027	0.022
F80+	0.071	0.268	0.168	0.117	0.085	0.064	0.048	0.037	0.029	0.023	0.018
M80+	0.099	0.229	0.149	0.107	0.081	0.063	0.05	0.04	0.032	0.026	0.021

SHI means social health insurance, F and M denote female and male, respectively

The results for group with SHI revealed the same pattern of using healthcare utilization as those of group without SHI. A possible explanation for such findings on gender is that although women, on average, live longer than men do, they tend to have poor health than men. By contrast, men tend to have chronic diseases that are associated with higher rates of mortality than do women. Thus, at low count events (e.g., count 1–4), women may use more healthcare utilization than men, but men may need more healthcare at higher count events (e.g., count 5–10) due to the severity of their health conditions. As for comparison of groups with and without SHI, at count 1–4 people without SHI had more healthcare utilization than those with SHI. However, the results showed a reverse trend at count 5–10. This finding could be partly explained by medical costs for outpatient services as high medical costs could present a huge barrier for people without SHI to access to healthcare, especially at high count events.

## Discussion and conclusions

This study has showed strong evidence of overdispersion, but no substantial excess of zeros in the outpatient visits data for Viet Nam’s older people. Consequently, the PRM performed the worst among the count models considered and its extensions also showed poor fit. Literature has shown that ignoring the overdispersion issue leads to deflated standard errors and inflated *z*-values, although estimates of parameters from the PRM are still consistent even when the equidispersion property is violated [5, 13, 14]. Although the ZINB2 model had a convergence problem, its results showed a better fit than those of the PRM and its extensions. In this study, the same set of explanatory variables are used to model both structural and sampling zeros, which could be a reason for the convergence problems with the ZINB2 model [25, 26]. The results of in-sample selection showed that the NBRM fit the data better than did the ZIM. Among the NBRM, the NB2 model was preferred over its NB1 counterpart.

The assumption of the NBRM that the process of generating zeros is the same as that of positive observations has been criticized as too restrictive in the modeling of healthcare utilization [1]. Critics argue that the decision to initiate the first contact to a doctor and the subsequent visits may be made in two different processes. As such, the LCNB2 undoubtedly fit the data better than did the NBRM. However, the in-sample selection results showed that the HNB beat all other count models considered. Among the HNB, the HNB2 fit the data better than did the HNB1.

The results of 10-fold cross-validation showed that the HNB2 was the best-fitted model in most replications. Comparison among regression models for healthcare utilization and other fields has been widely conducted, though the results are mixed in the literature. Deb and Norton [23] find that the HNB model is more appropriate than the PRM and the NBRM for estimation of office-based visits, while the NBRM best fits the data for emergency department visits. Regarding comparison of the HRM and LCM, Jiménez-Martín et al. [28] find that the LCM is preferred in the case of general practitioners, while the TPM fits the data better than the LCM when count outcome is number of visits to specialists. Cameron and Trivedi [5], using a recreational trips data set for comparison of the HNB and LCM, find that the HNB fits that data better than does the LCM. In line with Cameron and Trivedi’s work, Winkelmann, who uses Poisson-log-normal in the second part of the HRM, shows that the HNB describes the number of doctor visits better than does the LCM [29]. Findings of this study are in agreement with the findings of those studies, but in contrast with those of studies by Deb and Trivedi [4, 26] and Sarma and Simpson [27], which find strong evidence in favor of the LCM relative to the HNB.

The results of marginal effects showed that Kinh people had more outpatient visits than non-Kinh people. A possible explanation is barriers of access to healthcare among minority people due to long commutes to health institutions in Vietnam [32]. People from larger families had fewer outpatient visits than those from smaller families. This finding is inconsistent with that of Deb and Trivedi [4]. There is no clear explanation for such finding, although we speculate that it may indicate unobserved financial stress, i.e., people from larger families may choose not to go to a hospital because of lack of finances, even though a certain level of healthcare is needed. Mixed results are found for region of residence, with both positive and negative effects on number of outpatient visits. This result is consistent with previous studies, indicating that healthcare utilization varies by sub-regions [4, 27]. Having SHI had a positive and significant effect on the number of outpatient visits, implying that having SHI leads to an increase in demand for outpatient visits. A possible explanation could be the *ex-ante* moral hazard in healthcare utilization for non-hospitalized services, i.e., people with SHI tend to use more outpatient services because they know that insurance companies bear part of the cost for such services. Findings of this study are in agreement with previous studies, showing that having SHI or supplemental health insurance increases individuals’ healthcare utilization [5, 30, 31].

Healthcare utilization is responsive to need factors, measured by NCDs and disability. Particularly, having at least one NCD or disability increased the number of outpatient visits. This seems reasonable since most NCDs or disabilities require care management, rather than hospitalization (with the exception of severe conditions). Similar findings have been found in the literature [1, 4, 30]. As for lifestyle variable, smoking had a negative effect on number of outpatient visits and this finding contrasts with that of Sarma and Simpson [27].

The study has some limitations, primarily arising from the nature of the data. First, this is a cross-sectional study, so it cannot provide any causal analysis between the determinants and healthcare utilization. Second, the cross-sectional study, moreover, only captures whether a person used outpatient services at the time of the survey conducted, thus we don’t know how individuals’ healthcare utilization behaviors change over times. A longitudinal study is needed to observe such changes in healthcare utilization behaviors. Third, we acknowledge the possibility of recalled bias, since the count outcome used in this study was based on self-reported information. Finally, this study used the 2006 VHLSS to demonstrate our empirical econometric strategies, thus the findings of this study may not reflect the current trend of healthcare utilization of Vietnamese older people.

Despite those limitations, this study’s findings lay the groundwork for future research on the modeling of healthcare utilization in developing countries, and those findings could be used to forecast healthcare demand and making provisions for healthcare costs. The factors associated with number of outpatient visits and the trends of healthcare utilization among specific groups could serve to inform policy and guide public health interventions to mitigate inequity in healthcare utilization for a rapidly aging population. Although older women, on average, tend to have poorer health than men, this study showed that the intensive margins were higher for men than it was for women, suggesting that policy should target not only women, but also men. Improvement in count data in the future is essential to provide an accurate understanding of the associated factor with healthcare utilization. Some other important variables are encouraged to be included in future surveys such as detailed types of SHI or complemental health insurance, waiting time, travel time, the number of the number of visits to a general health professional, the number of visits to a specialist, and the number of nights spent as a hospital patient. Such variables could provide interesting insights and point to essential parameters related to healthcare utilization.

Healthcare utilization data come in different forms and the high degree of skewness and dispersion that typically characterizes such data affects the appropriateness of the econometric models that should be used in modeling such data. It is hard to conclude one model is superior to others. On one hand, the HNB may fit a given dataset better if the data-generating process reflects the actual two different decision-making processes (whether to use healthcare or not, and conditional on the decision of use of healthcare, how much care to consume). On the other hand, researchers may select LCM for a given dataset if latent heterogeneity divides the population into classes that may respond differently to changes in covariates. Our findings suggest that HNB2 was the best-fitting model and that it should be considered for modeling healthcare utilization in other contexts with similar characteristics as the population examined in this study. Nevertheless, researchers are encouraged to deeply investigate alternative models to avoid model misspecification.

## Data Availability

The data underlying this study were provided by the Vietnam General Statistics Office. Data can be accessed by applying through the Vietnam General Statistics Office at https://www.gso.gov.vn/en/homepage/, or contacting Mr. Nguyen The Quan at ntquan@gso.gov.vn.

## Abbreviations

PRM: The Poisson regression model
NBRM: The negative binomial regression model
NB1: The negative binomial 1
NB2: The negative binomial 2
HRM: Hurdle regression model
HNB1: Hurdle negative binomial 1
HNB2: Hurdle negative binomial 2
HPM: Hurdle Poisson model
ZIM: Zero-inflated regression model
ZINB2: Zero-inflated negative binomial 2
LCM: Latent class model
LCNB2: Latent class negative binomial 2
SHI: Social health insurance
OOP: Out-of-pocket
VHLSS: Vietnam Household Living Standard Survey
NCDs: Non-communicable diseases
LR: Likelihood ratio
AIC: The Akaike information criteria
BIC: The Bayesian information criteria
RMSE: Root mean square error
MAPE: Mean absolute prediction error
